# Retraction Note: Novel life history strategy in a deep sea fish challenges assumptions about reproduction in extreme environments

**DOI:** 10.1038/s41598-020-75408-8

**Published:** 2020-10-29

**Authors:** Randal A. Singer, Jon A. Moore, Edward L. Stanley

**Affiliations:** 1 University of Michigan Museum of Zoology, University of Michigan, Ann Arbor, MI 48108 USA; 2 Florida Atlantic University, Wilkes Honors College, 5353 Parkside Drive, Jupiter, FL 33458 USA; 3 Florida Museum of Natural History, University of Florida, Gainesville, FL 32611–7800 USA

Retraction of: *Scientific Reports* 10.1038/s41598-020-60534-0, published online 27 February 2020.

The authors have retracted this Article. It has been brought to the Authors' attention^1^ that the eggs found in the buccal cavity of the examined specimen may actually be those of a species of crab. Re-analysis of the original samples confirmed this to be the case. While the breeding ecology of *Parazen pacificus* remains unknown and the species appears to be exapted for mouthbrooding, we find that the original data is not sufficient to confirm that this species is a mouth brooder.

All authors agree with the retraction.
